# No significant drug−drug interaction between oral TAF‐based PrEP and feminizing hormone therapy among transgender women in Thailand: the iFACT‐3 study

**DOI:** 10.1002/jia2.26502

**Published:** 2025-05-19

**Authors:** Akarin Hiransuthikul, Narukjaporn Thammajaruk, Stephen Kerr, Rena Janamnuaysook, Siriporn Nonenoy, Piranun Hongchookiat, Rapee Trichavaroj, Yardpiroon Tawon, Jakkrapatara Boonruang, Nipat Teeratakulpisarn, Tim R. Cressey, Peter L. Anderson, Nittaya Phanuphak, the iFACT3 study team

**Affiliations:** ^1^ Institute of HIV Research and Innovation (IHRI) Bangkok Thailand; ^2^ Department of Preventive and Social Medicine Faculty of Medicine Chulalongkorn University Bangkok Thailand; ^3^ Biostatistics Excellence Centre, Faculty of Medicine Chulalongkorn University Bangkok Thailand; ^4^ HIV‐NAT, Thai Red Cross AIDS Research Centre Bangkok Thailand; ^5^ The Kirby Institute University of New South Wales Sydney New South Wales Australia; ^6^ Center of Excellence in Transgender Health (CETH) Faculty of Medicine Chulalongkorn University Bangkok Thailand; ^7^ AMS/PHPT Research Collaboration Faculty of Associated Medical Sciences Chiang Mai University Chiang Mai Thailand; ^8^ Department of Pharmaceutical Sciences University of Colorado, Anschutz Medical Campus Aurora Colorado USA

**Keywords:** drug−drug interactions, feminizing hormone therapy, HIV prevention, pre‐exposure prophylaxis, Thailand, transgender women

## Abstract

**Introduction:**

Concerns regarding potential drug−drug interaction (DDI) between feminizing hormone therapy (FHT) and HIV pre‐exposure prophylaxis (PrEP) may hinder PrEP use among transgender women. We assessed the potential DDI between FHT and emtricitabine‐tenofovir alafenamide (F/TAF)‐based PrEP among transgender women.

**Methods:**

Transgender women without HIV who never underwent orchiectomy were enrolled between January and February 2022. Oral FHT (oestradiol valerate 2 mg and cyproterone acetate 25 mg) was initiated at baseline and continued until week 9, while oral PrEP (F/TAF 200/25 mg) was initiated at week 3 and continued until week 12. Intensive blood sampling was performed at weeks 3 and 9 to assess the impact of PrEP on FHT; and weeks 9 and 12 to assess the impact of FHT on PrEP. Pharmacokinetics (PKs) of plasma oestradiol (E2), TAF, tenofovir (TFV) and emtricitabine (FTC); urine TFV and FTC; and tenofovir‐diphosphate (TFV‐DP) and emtricitabine‐triphosphate (FTC‐TP) in peripheral blood mononuclear cells (PBMCs) and rectal tissues were assessed.

**Results:**

Eighteen participants completed all PK visits. No significant differences in PK parameters for plasma E2, TAF and TFV were observed with FHT and F/TAF administration. The geometric mean of FTC AUC_0−24_ at week 9 was 9% lower than at week 12, but the 90% CI (0.88−0.95) remained within the 80–125% range. There were no significant differences in PBMCs and rectal tissues TFV‐DP and FTC‐TP concentrations when F/TAF was administered with FHT.

**Conclusions:**

No bidirectional clinically significant DDI between FHT and F/TAF‐based PrEP was observed across systemic and local tissue anatomical compartments, supporting the use of oral F/TAF‐based PrEP among transgender women.

**Clinical Trial Number:**

NCT04590417

## 1 INTRODUCTION

Transgender women face a disproportionate burden of HIV, with an estimated prevalence of 19.1% and a 49‐fold increased odds of HIV acquisition compared to the general population [1].

Among challenges such as stigma and negative experiences with providers, clients’ prioritization of gender‐affirmative interventions, including hormone therapy, over pre‐exposure prophylaxis (PrEP) presents a major challenge contributing to PrEP underutilization [2, 3].

Feminizing hormone therapy (FHT) is commonly used among transgender women to induce the secondary sex characteristics of the affirmed gender and to reduce the sex characteristics of the sex designated at birth [4, 5]. This involves the concurrent use of exogenous oestrogens and anti‐androgenic medications (both prescription and off‐label) with various regimens recommended by expert guidance [6]. In Thailand, up to 75% of transgender women report using FHT for significant periods (mean duration of 10 years), often at doses exceeding recommended doses [7]. While concern about the potential negative impact of PrEP on the efficacy of FHT has been consistently raised as barriers to PrEP uptake and adherence among transgender women [3, 8], studies have demonstrated no significant difference in oestradiol (E2) pharmacokinetic (PK) parameters with or without concomitant use of emtricitabine‐tenofovir disoproxil fumarate (F/TDF)‐based PrEP use [9, 10, 11, 12].

The use of daily oral F/TDF for PrEP is effective in reducing HIV transmission among men who have sex with men (MSM), heterosexual men and women, and people who inject drugs [13, 14, 15]. However, few transgender women were included in the primary PrEP trials, and there were also concerns about potential negative drug−drug interactions (DDIs) between F/TDF‐based PrEP and FHT. While studies, including our own trial, have shown a slight negative impact of FHT on the PK parameters of F/TDF‐based PrEP among transgender women [10, 16–18], the observed reduction in tenofovir concentrations remains within the target range and is unlikely to affect the efficacy of oral F/TDF‐based PrEP.

Tenofovir alafenamide (TAF) is a newer prodrug formulation of tenofovir that achieves higher intracellular concentrations of tenofovir‐diphosphate (TFV‐DP), the active intracellular metabolite of tenofovir, in lymphatic tissue while having lower plasma tenofovir concentrations compared to TDF. The DISCOVER trial demonstrated that daily oral F/TAF‐based PrEP was statistically non‐inferior to F/TDF‐based PrEP in preventing HIV acquisition. PKs assessment revealed that intracellular TFV‐DP concentrations in peripheral blood mononuclear cell (PBMC) were 6.3‐fold higher with F/TAF compared to F/TDF [19]. A post hoc analysis of DISCOVER participants found comparable PBMC TFV‐DP and FTC‐TP concentrations between transgender women using FHT and cisgender MSM receiving F/TAF [20]. However, this analysis was limited by a small sample of transgender women and a single sampling time point.

Given that many transgender women are concerned about the impact of PrEP on their FHT, and the observed DDI between FHT and F/TDF‐based PrEP, it is important to understand the bidirectional effects of FHT on F/TAF‐based PrEP. These data are essential in providing evidence‐based guidance to transgender women about PrEP use with concomitant FHT. And since effective HIV prevention requires antiretroviral drugs to reach sufficiently high concentrations in target mucosal tissues and in PBMCs [21, 22], this study aimed to assess the potential DDI between FHT and daily oral F/TAF‐based PrEP among transgender women, utilizing drug measurements in systemic and local tissue anatomical compartments.

## 2 METHODS

### 2.1 Enrolment and study population

Between January and February 2022, transgender women attending the Tangerine Community Health Centre, Institute of HIV Research and Innovation, in Bangkok, Thailand were screened for enrolment. Thai transgender women without HIV aged 18–40 years, with body mass index (BMI) of 18.5−24.9 kg/m^2^, calculated creatinine clearance (CrCl) ≥60 ml/minute using Cockcroft−Gault equation and alanine aminotransferase (ALT) ≤2.5 × upper limit of normal were eligible. Participants with a history of allergy to the study hormonal compounds, who had undergone orchiectomy, had received injectable FHT in the previous 3 months, had current hepatitis B or C virus infection, were currently using a medication with possible DDI with FHT and/or PrEP, or had a history of gastrointestinal tract surgery that alter gastrointestinal tract were excluded. The target sample size was 20 transgender women. The study (NCT04590417) was approved by the institutional review board of the Faculty of Medicine, Chulalongkorn University, Bangkok, Thailand (IRB No. 728/63), and all participants provided informed consent prior to all study procedures.

### 2.2 Study drugs

The FHT regimen selected was oestradiol valerate and cyproterone acetate (CPA), which was in line with the Tangerine Community Health Centre's FHT protocol and consistent with the availability and popularity of FHT regimens in the local Thai transgender women community. This approach mirrored that used in our previous studies on DDI between FHT and antiretroviral agents [10, 23].

All participants were placed on the same FHT regimen comprising oral oestradiol valerate 2 mg (Progynova®, Delpharm Lille S.A.S., Lyz Lez Lannoy, France) and oral cyproterone acetate 25 mg (Androcur®, Bayer Weimar GmbH und Co. KG, Weimar, Germany) once daily for FHT. Daily oral F/TAF (Descovy®, Gilead Sciences, Foster City, CA, USA) was used for PrEP.

### 2.3 Study procedures

**Figure 1:**
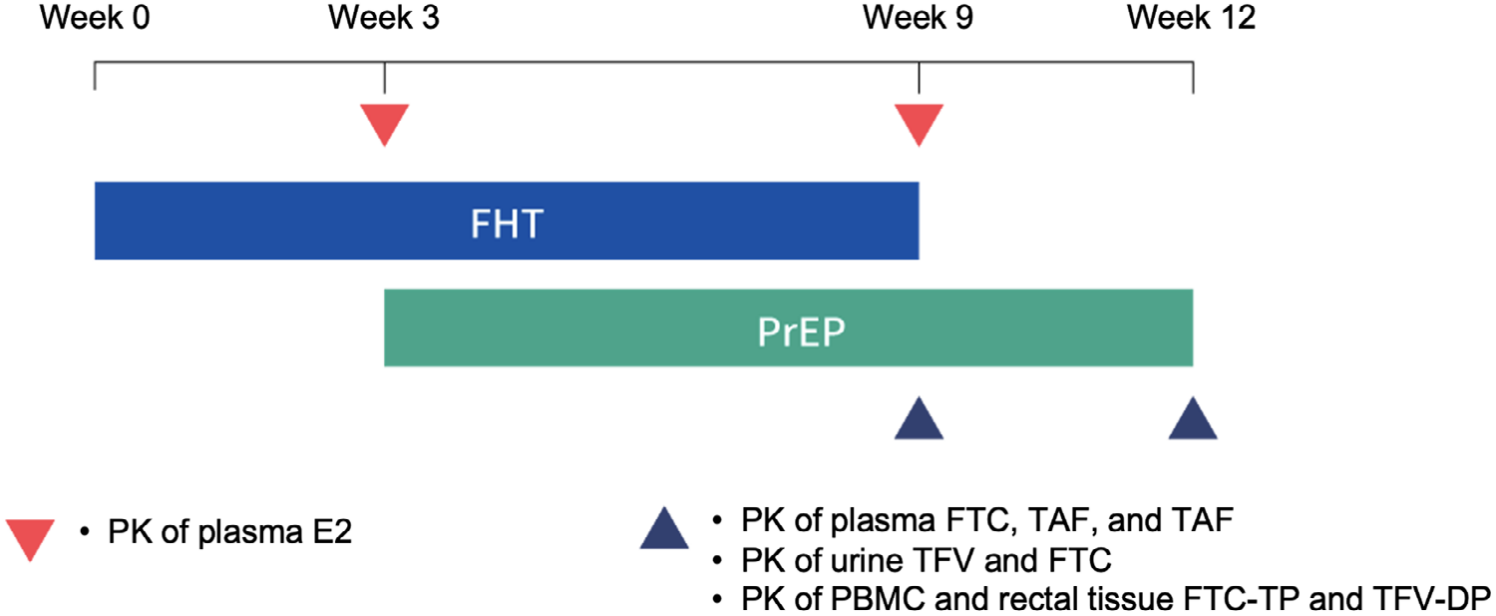
iFACT3 study scheme. Oral FHT was initiated at week 0 until week 9. Oral F/TAF‐based PrEP was initiated at week 3 and continued without interruption until the completion of study period at week 12. Intensive PK parameters of E2 were assessed at week 3 (FHT only) and week 9 (FHT + PrEP). Intensive PK parameters of ARVs were assessed at week 9 (FHT + PrEP) and week 12 (PrEP only). Abbreviations: ARVs, antiretroviral; E2, oestradiol; FHT, feminizing hormone therapy; FTC, emtricitabine; FTC‐TP, FTC‐triphosphate; PBMC, peripheral blood mononuclear cell; PK, pharmacokinetic; PrEP, pre‐exposure prophylaxis; TAF, tenofovir alafenamide; TFV, tenofovir; TFV‐DP, TFV‐diphosphate.

To assess the impact of oral F/TAF PrEP on FHT, and vice versa, intensive PK sampling was performed at three distinct time points: (1) participants receiving FHT only; (2) participants receiving FHT with PrEP; and (3) participants receiving PrEP only, conducted in this chronological order. FHT was prescribed to participants at entry and then the first PK sampling for oestradiol (E2) was performed at week 3. Afterwards, daily oral F/TAF PrEP was initiated. At week 9, the second intensive PK sampling for E2 and F/TAF was conducted. FHT was then discontinued and the third intensive PK sampling for F/TAF was performed at week 12 (Figure 1). Comparisons between PK parameters at week 3 and week 9 would provide information on the potential impact of PrEP on FHT, while the comparisons between week 9 and week 12 would provide information on the potential impact of FHT on PrEP. The sequence was selected to prioritize consistent PrEP use and avoiding any temporary discontinuation.

To ensure adherence to study drugs and prevent external FHT and PrEP, electronic directly observed therapy and hormone measurements were utilized. The study staff contacted participants daily, either through video calls or messaging applications, to confirm their adherence to the study drug. Additionally, pill counts and self‐reported use of other medications were assessed at each visit.

### 2.4 PK sampling

A 24‐hour PK assessment was performed for plasma E2, TAF, TFV and FTC. Plasma samples were collected at t = 0 (pre‐dose), and then 0.5, 1, 2, 4, 6, 8, 10, 12 and 24 hours after a directly observed intake of study drugs with a standardized meal (total of 10 samples). The focus was on the time points close to the initial dosing to accurately capture the peak concentrations. PBMC samples were collected at t = 2 (C_2_) and 24 (C_24_) hours and all urine passed during the 24‐hour period was collected. Rectal tissues were obtained at the PK visit 16–24 hours post‐dose in a subset of 10 participants. Tissue pinch biopsies were weighed at the clinical site, flash frozen at −70°C and stored for analysis using a validated assay. Detailed specimen collection and processing procedures are available in the Supplementary File.

### 2.5 Measurement of hormonal response

Pre‐dose plasma samples for follicle‐stimulating hormone (FSH), luteinizing hormone (LH) and total testosterone assessment were collected at weeks 0, 3 and 9 to assess hormonal response in the absence and presence of PrEP. Bioavailable testosterone was calculated from total testosterone using a previously reported method [24]. Hormone concentrations at week 12 were also collected to serve as biological markers to evaluate participants’ adherence to the protocol.

### 2.6 Statistical analysis

Sample size calculations were performed using the two one‐sided tests procedure of Schuirmann [25]. We designed the study to have sufficient power to determine whether the 90% confidence interval (CI) for the geometric mean ratio (GMR) of the area under the plasma concentration time curve from time 0 to 24 hours (AUC_0−24_) and maximum concentration (C_max_) for either the FHT or PrEP, when given alone versus in combination, fell outside the bioequivalence range of 0.8−1.25, as a surrogate of clinically significant change. Assuming the coefficients of variation for the FHT and PrEP given alone and in combination were 0.45 and 0.35, respectively, enrolling 20 participants would give 80% power to detect that the 90% CI around the GMR of the AUC and C_max_ for drugs given alone versus in combination were <80% of >125%.

Statistical and PK analyses were performed using Stata/SE 17.0 (StataCorp, College Station, TX, USA). AUC_0−24_ and C_max_ were derived using non‐compartmental models to assess the PK of E2, TAF, TFV and FTC. The geometric mean (GM) and % coefficient of variation (%CV) were summarized at each study week. Generalized estimating equations were used to assess the change in the AUC_0−24_ and C_max_ GM to calculate the GMR for plasma E2, TAF, TFV and FTC; PBMC TFV‐DP and FTC‐TP; and urine TFV and FTC; the reference week was the point at which the FHT or PrEP was administered alone. Rectal tissues TFV‐DP and FTC‐TP were reported with median (IQR) at each study week and compared with a Wilcoxon sign‐rank test. Consistent with the US FDA Guidelines for Industry on Drug Interaction Studies, the 90% CI around the GMR was calculated, with a target range of 80−125% to demonstrate bioequivalence and indicate no clinically significant differences. Changes in bioavailable testosterone and other laboratory parameters from baseline to subsequent study weeks were tested with a Wilcoxon sign rank test.

## 3 RESULTS

Twenty transgender women without HIV were enrolled. Two participants were excluded from the analysis due to loss to follow‐up. The baseline characteristics of all 18 participants are provided in Table 1. All participants had prior experience with FHT, maintained 100% adherence to both FHT and PrEP per the study protocol, and none acquired HIV during the study period.

**Table 1:** Participant characteristics at baseline study visit

Characteristic	Median (IQR)
Age (years)	28 (23–32)
Weight (kg)	60.3 (55.8–64.9)
Height (cm)	172 (168–175)
BMI (kg/m^2^)	20.8 (19.9–21.9)
CrCl (ml/minute)	125.0 (101.5–137.0)
ALT (U/l)	18.0 (13.1–25.5)

Abbreviations: ALT, alanine aminotransferase; BMI, body mass index; CrCl, creatinine clearance.

### 3.1 PK parameters of E2 and F/TAF

#### 3.1.1 Plasma E2

**Figure 2:**
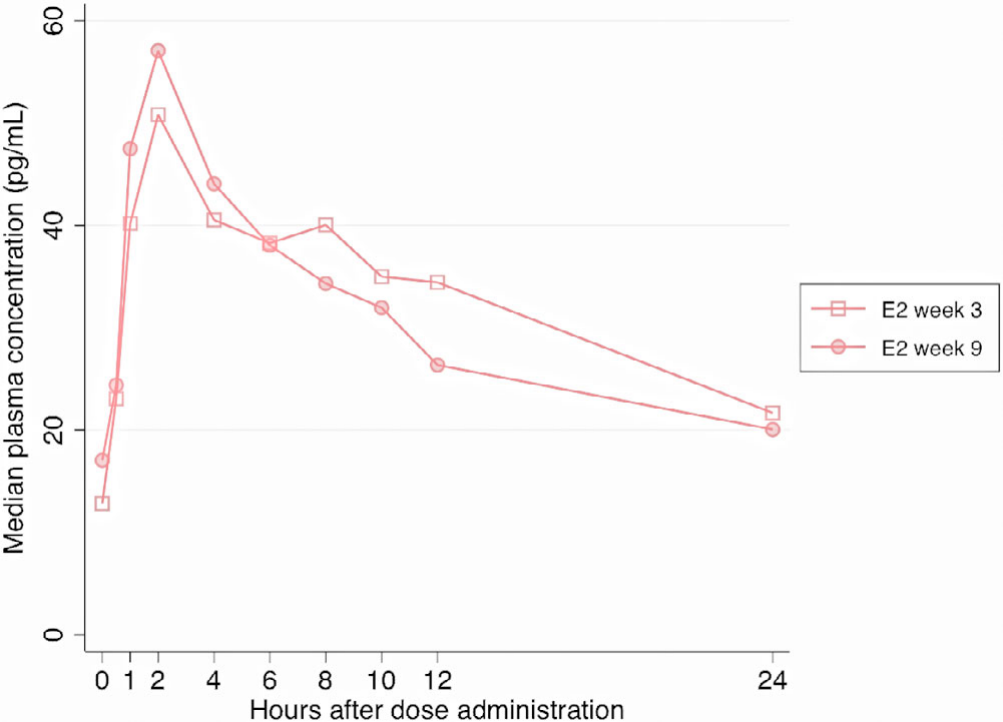
Median concentration time curves of plasma E2 at week 3 (without PrEP) and week 9 (with PrEP). Undetectable E2 (<5 pg/ml) imputed as 2.5 pg/ml. Abbreviation: E2, oestradiol.

The E2 AUC_0−24_ and C_max_ GMRs at week 3 (reference) and week 9 were 0.98 (0.91−1.05, *p* = 0.70) and 1.11 (0.96−1.27, *p* = 0.23), respectively (Table 2). The median blood plasma concentration‐time profiles of E2 are shown in Figure 2.

**Table 2:** Geometric mean (%CV) pharmacokinetic parameters for plasma E2, with and without PrEP

E2 PK parameter	Week 3 (without PrEP)	Week 9 (with PrEP)	GMR (90% CI)	*p*‐value
AUC_0−24_ (pg*hour/ml)	799.41 (35.63)	782.47 (36.91)	0.98 (0.91–1.05)	0.70
C_max_ (pg/ml)	60.13 (34.46)	66.59 (39.78)	1.11 (0.96–1.27)	0.23

Abbreviations: %CV, percentage coefficient of variation; AUC_0–24_, area under curve from time 0 to 24 hours; CI, confidence interval; C_max_, maximum concentration; E2, oestradiol; GMR, geometric mean ratio; PK, pharmacokinetic; PrEP, pre‐exposure prophylaxis.

#### 3.1.2 Plasma TAF, TFV and FTC

**Figure 3:**
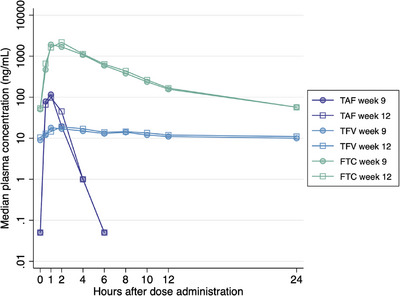
Median concentration time curves of plasma TAF, TFV and FTC at week 9 (with FHT) and week 12 (without FHT). Undetectable TAF imputed as 0.05 ng/ml. Abbreviations: FHT, feminizing hormone therapy; FTC, emtricitabine; TAF, tenofovir alafenamide; TFV, tenofovir.

The AUC_0−24_ and C_max_ GMRs at week 9 and week 12 (reference) were as follows: TAF, 1.05 (0.83−1.33, *p* = 0.78) and 1.14 (0.85−1.52, *p* = 0.55); TFV, 0.93 (0.89−0.98, *p* = 0.07) and 0.97 (0.89−1.05, *p* = 0.58); and FTC, 0.91 (0.88−0.95, *p* = 0.001) and 0.93 (0.84−1.03, *p* = 0.33), respectively (Table 3). The GM of FTC AUC_0−24_ at week 9 was statistically significantly less than that at week 12 by 9%, but the 90% CI around the GMR still fell inside the 80–125% boundary. All participants had quantifiable TFV and FTC concentrations in both visits. The median plasma concentration‐time profiles of F/TAF drug components are shown in Figure 3.

**Table 3:** Geometric mean (%CV) pharmacokinetic parameters for plasma TAF, TFV and FTC

ARV PK parameter	Week 9 (with FHT)	Week 12 (without FHT)	GMR (90% CI)	*p*‐value
TAF				
AUC_0−24_ (ng*hour/ml)	208.09 (65.50)	197.88 (50.04)	1.05 (0.83–1.33)	0.78
C_max_ (ng/ml)	166.78 (90.76)	146.65 (56.84)	1.14 (0.85–1.52)	0.55
TFV				
AUC_0−24_ (ng*hour/ml)	295.01 (18.43)	319.75 (16.25)	0.93 (0.89–0.98)	0.07
C_max_ (ng/ml)	19.47 (24.37)	20.17 (16.79)	0.97 (0.89–1.05)	0.58
FTC				
AUC_0−24_ (ng*hour/ml)	10,418.01 (15.17)	11,432.30 (13.25)	0.91 (0.88–0.95)	0.001
C_max_ (ng/ml)	2197.23 (33.00)	2366.24 (23.17)	0.93 (0.84–1.03)	0.33

Abbreviations: %CV, percentage coefficient of variation; ARV, antiretroviral; AUC_0–24_, area under curve from time 0 to 24 hours; CI, confidence interval; C_max_, maximum concentration; FHT, feminizing hormone therapy; FTC, emtricitabine; GMR, geometric mean ratio; PK, pharmacokinetic; TAF, tenofovir alafenamide; TFV, tenofovir.

#### 3.1.3 Urine TFV and FTC

The urine TFV and FTC GMRs at week 9 and week 12 were 1.05 (0.87−1.27, *p* = 0.73) and 0.92 (0.77−1.09, *p* = 0.50), respectively. There were no statistically significant differences in urine TFV and FTC concentrations between the 2 weeks.

#### 3.1.4 PBMC TFV‐DP and FTC‐TP

The PBMC C_2_ and C_24_ GMRs at week 9 and week 12 were as follows: TFV‐DP, 1.04 (0.93−1.16, *p* = 0.67) and 0.96 (0.84−1.10, *p* = 0.71); and FTC‐TP, 0.97 (0.87−1.08, *p* = 0.68) and 0.91 (0.77−1.07, *p* = 0.42), respectively (Table 4). No statistically significant differences in both PK parameters of TFV‐DP and FTC‐TP concentrations in PBMC between the 2 weeks were observed. All participants had TFV‐DP concentrations well above the 90% HIV prevention efficacy threshold (EC_90_) of >16 fmol/10^6^ PBMCs at both visits [21]. The lowest observed C_24_ were 255.35 fmol/10^6^ PBMCs at week 9 and 201.11 fmol/10^6^ PBMCs at week 12 (Figure S1).

**Table 4:** Geometric mean (%CV) pharmacokinetic parameters for PBMC TFV‐DP and FTC‐TP

ARV PK parameter (fmol/million cells)	Week 9 (with FHT)	Week 12 (without FHT)	GMR (90% CI)	*p*‐value
TFV‐DP				
C_2_	528.47 (28.16)	509.2 (30.02)	1.04 (0.93–1.16)	0.67
C_24_	427.07 (27.16)	443.77 (32.42)	0.96 (0.84–1.10)	0.71
FTC‐TP				
C_2_	6044.03 (37.86)	6244.14 (33.67)	0.97 (0.87–1.08)	0.68
C_24_	3492.81 (38.06)	3850.55 (45.38)	0.91 (0.77–1.07)	0.42

Abbreviations: %CV, percentage coefficient of variation; ARV, antiretroviral; C_2_, concentration at 2 hours; C_24_, concentration at 24 hours; CI, confidence interval; FHT, feminizing hormone therapy; FTC‐TP, emtricitabine‐triphosphate; GMR, geometric mean ratio; PK, pharmacokinetic; TFV‐DP, tenofovir‐diphosphate.

#### 3.1.5 Rectal tissue TFV‐DP and FTC‐TP

Among the 10 participants who underwent rectal tissue biopsies, there were no statistically significant differences in TFV‐DP and FTC‐TP concentrations in rectal tissue between weeks 9 and 12 (Table 5 and Figure S2). All participants had quantifiable TFV‐DP and FTC‐TP concentrations at both visits.

**Table 5:** Median (IQR) pharmacokinetic parameters for rectal tissue TFV‐DP and FTC‐TP (*N* = 10)

ARV PK parameter (fmol/mg)	Week 9 (with FHT)	Week 12 (without FHT)	*p*‐value
TFV‐DP	37.62 (21.41–45.79)	27.42 (21.44–56.27)	0.72
FTC‐TP	14.51 (12.39–17.02)	11.61 (8.04–14.48)	0.20

Abbreviations: ARV, antiretroviral; FHT, feminizing hormone therapy; FTC‐TP, emtricitabine‐triphosphate; IQR, interquartile range; PK, pharmacokinetic; TFV‐DP, tenofovir‐diphosphate.

### 3.2 Other hormonal responses: bioavailable testosterone, FSH and LH

Median (IQR) plasma concentrations of bioavailable testosterone, FSH and LH were comparable between week 3 and week 9 (bioavailable testosterone: 0.03 [0.03−0.12] vs. 0.02 [0.01−0.12] ng/ml, *p* = 0.17; FSH: 0.8 [0.6−1.3] vs. 0.9 [0.4−1.6] IU/l, *p* = 0.24; and LH: 0.52 [0.21−0.86] vs. 0.44 [0.25−0.79] IU/l, *p* = 0.95).

### 3.3 Hormone concentrations for adherence assessments

Following the discontinuation of FHT at week 9, there was a significant increase in bioavailable testosterone, FSH and LH concentrations. The differences in median (IQR) plasma concentrations of bioavailable testosterone, FSH and LH between weeks 9 and 12 were as follows: bioavailable testosterone, 0.02 (0.01−0.12) versus 2.1 (1.7−3.2) ng/ml, *p*<0.001; FSH, 0.9 (0.4−1.6) versus 6.2 (3.5–8.5) IU/l, *p*<0.001; and LH, 0.4 (0.3−0.8) versus 4.7 (2.9−6.7) IU/l, *p*<0.001.

### 3.4 Safety

No study‐related adverse events were reported during the study period. The median (IQR) CrCl at entry and at weeks 3, 9 and 12 was 125.0 (101.5–137.0), 117.8 (103.4–128.8), 118.1 (102.8–124.8) and 116.0 (99.1–125.5) ml/minute, respectively. The median ALT levels were 18.0 (13.1–25.5), 13.8 (12.0–23.4), 14.5 (12.0–22.2) and 16.1 (10.8–19.8) U/l, respectively. No significant changes in CrCl or ALT levels were observed from entry to the end of the study.

## 4 DISCUSSION

Our study assessed the impact of daily oral F/TAF‐based PrEP on FHT, and vice versa, among Thai transgender women. No significant changes in plasma E2 were observed when FHT was administered with and without PrEP. There were no significant changes in the PK of F/TAF components in plasma, urine, PBMC and rectal tissue compartments. Additionally, the concentrations of plasma bioavailable testosterone, FSH and LH remained comparable when PrEP was administered. These findings demonstrate no clinically significant DDI between FHT and F/TAF‐based PrEP.

Concerns regarding potential DDIs between FHT and PrEP have been highlighted as key barriers to the uptake and adherence of PrEP among transgender women [3, 8], particularly with the prioritization of FHT over PrEP, despite positive attitudes towards PrEP among this population [26, 27, 28]. However, given the distinct metabolic pathways of F/TAF components and FHT, clinically significant DDIs are not anticipated. Previous studies have shown no significant impact of F/TDF‐based PrEP on E2 among transgender women [9, 10, 11, 12]. The findings from our study, which utilized F/TAF‐based PrEP, further support the notion that transgender women using FHT can safely use all oral PrEP without concern for potential negative effects on the efficacy of FHT.

The impact of FHT on PrEP appears minimal, supporting its continued use for HIV prevention in transgender women. Although we observed a slight decrease in plasma FTC AUC_0−24_ when PrEP was administered with FHT, the 90% CI around the GMR still fell inside the 80–125% boundary, and the FTC‐TP concentration, the active intracellular metabolite, was not different [20, 29]. Similar to the post hoc analysis of DISCOVER participants [20], we found comparable PBMC TFV‐DP and FTC‐TP concentrations in the presence and absence of FHT. Notably, all participants had TFV‐DP concentrations well above the EC_90_ of >40 fmol/10^6^ PBMCs at both visits, providing strong support for the use of F/TAF for HIV prevention among transgender women using FHT.

The CD4‐bearing cells in rectal tissues are particularly relevant for HIV transmission, especially among individuals engaged in anal intercourse. Although two small studies found no statistical difference in rectal tissue TFV‐DP concentrations between transgender women and cisgender men, one study reported a seven‐fold reduction in the TFV‐DP:dATP ratio in transgender women [16, 17]. To our knowledge, no study has explored the potential DDI between FHT and F/TAF‐based PrEP using rectal tissue as the outcome. Our findings suggest no statistically significant differences in TFV‐DP and FTC‐TP concentrations in rectal tissue when administering F/TAF‐based PrEP with and without FHT, with all participants having quantifiable TFV‐DP and FTC‐TP concentrations at both visits.

Certain limitations need to be considered in our study. First, we did not use a randomized crossover design, so a period effect was possible. However, the study drugs’ half‐lives and the intervals between intensive PK sampling ensured that steady‐state conditions are achieved for each drug of interest, and that elimination of the drug dosed in the prior period was completed. Ideally, this PK study would have included FHT‐naive participants, but this was less feasible for recruitment. Nonetheless, non‐injectable FHT is expected to be eliminated from plasma by the first PK measurement at week 3. Issues with adherence to both continuation and discontinuation of the drugs, as well as the use of other FHT concurrently are possible. Nonetheless, we made every effort to ensure that participants adhered to the protocol by directly observing the study drugs administration in addition to using biological markers. For the FHT assessment, we were unable to measure CPA concentrations due to the unavailability of the laboratory test in our facility. To address this limitation, we opted to measure bioavailable testosterone concentrations as an indirect measurement of CPA activities. The use of a specific FHT regimen in this study, reflective of one of the most common regimens at the Tangerine Health Community Centre, may limit the generalizability of our findings to those using different FHT regimens. A potential limitation of our study design was that the single intensive PK sampling during each study period was insufficient to fully describe the intra‐patient variability. While an alternative design with sparse PK sampling repeated within each study period is reasonable, the intensive PK sampling design was deemed preferable to estimate both within‐ and between‐participant variability. The study blood/tissue sampling strategy (i.e. number/timing of samples, blood volumes, sample types) was also optimized for participant engagement, incorporating guidance from the community advisory board based on their feedback from participants in prior PK studies. For example, the invasiveness of rectal biopsies was thought to be a potential obstacle for recruitment and subsequently was only required in a subset of participants; while still ensuring that the number of biopsies collected was consistent with previous studies [16, 22]. Lastly, the nature of an intensive PK study design meant that all participants enrolled in our study had highly homogenous characteristics; therefore, the extent of a DDI between FHT and F/TAF‐based PrEP among transgender women could differ among diverse ethnicities, races, ages or BMI; although this was not the case for F/TDF [10, 16, 17].

## 5 CONCLUSIONS

Plasma E2 concentrations were unchanged in the presence of F/TAF‐based PrEP. Intracellular TFV‐DP and FTC‐TP concentrations in PBMC and rectal tissue were not significantly different when F/TAF‐based PrEP was administered with or without FHT. These findings suggest no bidirectional clinically significant DDI between FHT and F/TAF‐based PrEP in transgender women.

## COMPETING INTERESTS

All authors declare no competing interests related to this work.

## AUTHORS’ CONTRIBUTIONS

AH drafted the manuscript and study protocol. AH and NP initiated the study concept and led the study. NT and SN coordinated the study operations. AH, SK and RJ contributed to the study design. RJ and NP facilitated the grant coordination for the study. AH and SK conducted all statistical analyses. RT, YA, TRC and PLA performed the laboratory testing. NT, PH, JB and NT oversaw the participants. All authors critically reviewed and approved the final draft of the manuscript.

## FUNDING

This study was supported by an Investigator Sponsored Research (ISR) grant through Gilead Sciences (IN‐US‐412‐5796).

## Supporting information




**Figure S1**. Median concentrations of PBMC TFV‐DP and FTC‐TP, with plots of all participants at week 9 (with FHT) and week 12 (without FHT).
Abbreviations: C_2_, concentration at 2 hours; C_24_, concentration at 24 hours; FHT, feminizing hormone therapy; FTC‐TP, emtricitabine‐triphosphate; PBMC, peripheral blood mononuclear cell; TFV‐DP, tenofovir‐diphosphate.

**Figure S2**. Median concentrations of rectal tissue TFV‐DP and FTC‐TP, with plots of all participants at week 9 (with FHT) and week 12 (without FHT).
Abbreviations: FHT, feminizing hormone therapy; FTC‐TP, emtricitabine‐triphosphate; PBMC, peripheral blood mononuclear cell; TFV‐DP, tenofovir‐diphosphate.

**Supplementary File**. Details of specimen collection and processing procedure.

## Data Availability

The data that support the findings of this study are available from the corresponding author upon reasonable request.
