# Supplementary material for: No significant drug−drug interaction between oral TAF‐based PrEP and feminizing hormone therapy among transgender women in Thailand: the iFACT‐3 study
Source: J Int AIDS Soc. 2025 May 19;28(5):e26502. doi: 10.1002/jia2.26502 (PMC12089648; doi:10.1002/jia2.26502)
Supplement: Supplementary file 3 — Supplementary File. Details of specimen collection and processing procedure. [file JIA2-28-e26502-s002.docx]

**Supplementary File. Details of specimen collection and processing procedure**

Pharmacokinetic analysis of plasma estradiol

Clotted blood was collected and sent immediately to the IHRI laboratory for serum separation. Blood samples were centrifuged at 2,000 g for 10 min at ambient (18-25°C). Serum was separated and transferred to labeled polypropylene tubes and stored at -20°C until analysis. Estradiol was performed by COBAS e411 (Roche Diagnostics) based on the Electrochemiluminescence immunoassay (ECLIA) technique. The lower and upper limits of detection were 5 and 3,000 pg/mL, respectively (18.4-11,010 pmol/L).

Pharmacokinetic analysis of plasma total testosterone

EDTA blood was collected and sent immediately to the IHRI laboratory for serum separation. Blood samples were centrifuged at 2,000 g for 10 min at ambient (18-25°C). Plasma was separated and transferred to labeled polypropylene tubes and stored at -20°C until analysis. Total testosterone was performed by COBAS e411 (Roche Diagnostics) based on the Electrochemiluminescence immunoassay (ECLIA) technique. The lower and upper limits of detection were 0.025 and 15.0 ng/mL, respectively (0.087-52.0 nmol/L).

Pharmacokinetic analysis of plasma tenofovir alafenamide (TAF)

TAF was extracted from 100 μL of human plasma using protein precipitation with (v/v) formic acid in methanol containing the internal standard (Tenofovir Alafenamide-d5); the supernatant was added into a diluent (95% of 0.1% formic acid in water: 5% of 0.1% formic acid in methanol) and injected into the LC-MS/MS system.

Chromatographic separations were performed on a Synergy Polar reversed Phase C18 column (150 mm x 2.0 mm ID, particle size 4 µm), Phenomenex using a stepwise gradient at a flow rate of 0.3 mL/min. At time zero the flow consists of 1% mobile phase A (0.1% (v/v) formic acid in methanol) and 99% mobile phase B (0.1% (v/v) formic acid in water). The total run time was 18 min.

The standard calibration curve was linear over the range 2 to 500 ng/mL. The lower limit of quantification (LLOQ) was 2 ng/mL.

Accuracy ranged from 96% to 104%, with precision <6%.

Pharmacokinetic analysis of plasma tenofovir (TFV) and emtricitabine (FTC)

Human plasma (50 μL) was mixed with the internal standards (TFV-d6 and FTC-^13^C,^15^N_2_). Tenofovir (TFV), Emtricitabine (FTC) and its internal standards were extracted from the plasma using protein precipitation with 0.1% (v/v) formic acid in methanol then evaporate under nitrogen gas and reconstitution in a diluent (95% of 0.1% formic acid in water: 5% of 0.1% formic acid in methanol) and then injected into the LC-MS/MS system.

Chromatographic separations were performed on a Synergy Polar reversed Phase C18 column (150 mm x 2.0 mm ID, particle size 4um) using a stepwise gradient at a flow rate of 0.3 mL/min. At time zero the flow consists of 1% mobile phase A (0.1% (v/v) formic acid in methanol) and 99% mobile phase B (0.1% (v/v) formic acid in water). The total run time was 12 min.

The standard calibration curves were linear over the range 5 to 500 ng/mL for TFV and 5 to 2500 ng/mL for FTC. The lower limit of quantification (LLOQ) was 5 ng/mL for both analytes.

Accuracy ranged from 101% to 104% for TFV, and 98% to 102% for FTC, with precision <8% and <6% for TFV and FTC, respectively.

Pharmacokinetic analysis of urine TFV and FTC

Urine samples were prepared by making two separate dilutions: samples were first diluted 1:10 with DI water, and then diluted 1:10 with diluent (95% of 0.1% formic acid in water and 5% of 0.1% formic acid in methanol). 100 μL of the diluted sample wree mixed with 100 μL of IS working solution (TFV-d6 and FTC-^13^C,^15^N_2_), transferred into a HPLC vial, and then injected into the LC-MS/MS system.

Chromatographic separations were performed on a Synergy Polar reversed Phase C18 column (150 mm x 2.0 mm ID, particle size 4um) using a stepwise gradient at a flow rate of 0.3 mL/min. At time zero the flow consists of 1% mobile phase A (0.1% (v/v) formic acid in methanol) and 99% mobile phase B (0.1% (v/v) formic acid in water). The total run time was 12 min.

The standard calibration curve was linear over the range 100 to 10,000 ng/mL for TFV and 1,000 to 100,000 ng/mL for FTC. The lower limit of quantification (LLOQ) was 100 ng/mL for TFV and 1,000 ng/mL for FTC.

Accuracy ranged from 97% to 99% for TFV, and 98% to 101% for FTC, with precision <7% and <2% for TFV and FTC, respectively.

Pharmacokinetic analysis of peripheral blood mononuclear cells (PBMCs) TFV-diphosphate (TFV-DP) and FTC-triphosphate (FTC-TP)

PBMCs were lysed using 70% Methanol: 30% DI. The cell lysate was pipetted into the conditioned Waters QMA columns and washed with 75 mM and 90 mM KCl solutions, after which TFV-DP and FTC-TP are eluted with 1 M KCl. TFV-DP and FTC-TP are then incubated with alkaline phosphatase to dephosphorylate in a water bath at 37°C for 1 h. Afterwards, the isotopic internal standards were added (TFV-d6 and FTC-^13^C,^15^N_2_), and all samples desalted using 1M sodium acetate. Samples were then pipetted into the conditioned Stata-X cartridges, washed with 10 mM Ammonium Acetated and dichloromethane before being eluted with methanol. The methanol was evaporated under nitrogen gas and then reconstitution in a diluent (95% of 0.1% formic acid in water: 5% of 0.1% formic acid in methanol) and then injected into the LC-MS/MS system.

Chromatographic separations were performed on a Synergy Polar reversed Phase C18 column (150 mm x 2.0 mm ID, particle size 4um) using a stepwise gradient at a flow rate of 0.4 mL/min. At time zero the flow consists of 5% mobile phase A (0.1% (v/v) formic acid in methanol) and 95% mobile phase B (0.1% (v/v) formic acid in water). The total run time was 10 min.

The standard calibration curves were linear over the range 200 to 10,000 fmol/sample for TFV-DP and 500 to 100,000 fmol/sample for FTC-TP. The lower limit of quantification (LLOQ) is 200 fmol/sample for TFV-DP and 500 fmol/sample for FTC-TP.

Accuracy ranged from 92% to 104% for TFV-DP, and 95% to 108% for FTC-TP, with precision <10% and <4% for TFV-DP and FTC-TP, respectively.

Pharmacokinetic analysis of rectal tissue TFV-DP and FTC-TP

Tissue pinch biopsies were weighed at the clinical site and stored flash frozen at -70C prior to analysis. At the testing lab, tissues were transferred to homogenization tubes. 1 mL of cold (-20°C) 70:30 MeOH: 25 mM ammonium phosphate pH 7.4 was added and briefly vortexed to ensure all tissue was submerged in homogenizing solution. Samples were then homogenized using the Precellys homogenizer under cold conditions (-50°C using Cryolys) at 7500 rpm with 3 homogenization cycles for 30 seconds each with 30 second intermittent pause. Samples were then stored overnight at -80°C before another homogenization cycle was performed as before. Following homogenization, the sample was centrifuged and the supernatant transferred to a new 2 mL tube. This solution was assayed with a validated LC-MS/MS method, as described previously [1]. The analytical range was 25.0 - 6000 fmol/sample for TFV-DP and 0.1 – 200 pmol/sample for FTC-TP.

**Reference**:

1. Bushman LR, Kiser JJ, Rower JE, Klein B, Zheng JH, Ray ML, et al. Determination of nucleoside analog mono-, di-, and tri-phosphates in cellular matrix by solid phase extraction and ultra-sensitive LC-MS/MS detection. J Pharm Biomed Anal. 2011;56(2):390-401.
